# Synthesis and Characterization of Nano-Conducting Copolymer Composites: Efficient Sorbents for Organic Pollutants

**DOI:** 10.3390/molecules22050772

**Published:** 2017-05-10

**Authors:** Khadija M. Emran, Shimaa M. Ali, Aishah L. L. Al-Oufi

**Affiliations:** 1 Department of Chemistry, Faculty of Science, Taibah University, Madinah 30002, Saudi Arabia; k_and_e154@hotmail.com (K.M.E.); aloosh200933@hotmail.com (A.L.L.A.-O.); 2 Department of Chemistry, Faculty of Science, Cairo University, Giza 12613, Egypt

**Keywords:** nano-composite, copolymerization, characterization, adsorption, organic dye

## Abstract

Nano-conducting copolymers of aniline (ANI) and pyrrole (Py) with silica of different starting monomer ratios are prepared by oxidative chemical polymerization. X-ray diffraction (XRD) data showed that polyaniline (PANI) is the predominant phase in copolymer composites with a higher starting ANI monomer ratio while polypyrrole (PPy) is the major phase for other prepared samples. Transmission and scanning electron microscope images ascertained XRD results where hexagonal-shaped particles are assigned to PANI/SiO_2_ and poly(9ANI-co-1Py)/SiO_2_ samples; the cauliflower morphology can be observed for PPy/SiO_2_, poly(1ANI-co-9Py)/SiO_2_, poly(1ANI-co-2Py)/SiO_2_, and poly(1ANI-co-1Py)/SiO_2_ samples. One-dimensional nano-fibers can be obtained by using a starting monomer ratio of 2ANI:1Py during synthesis. Thermal analysis showed that copolymerization increases the thermal stability as compared with PANI/SiO_2_ and PPy/SiO_2_ composites. All prepared samples were applied as sorbents for Congo red dye from aqueous solutions. It was found that the sorption capacity value was affected by the starting monomer ratio; poly(2ANI-co-1Py)/SiO_2_ has the highest sorption capacity; the *q_m_* value is 142.9 mg g^−1^ due to its highly-stabilized nano-structure.

## 1. Introduction

Conducting polymers, such as polypyrrole (PPy), polythiophene (PT), polyaniline (PANI), polyfuran (PF), and derivatives, are conjugated π-electron systems that promote the charge-transfer process due to their considerable electrical conductivity and they offer many other interesting properties—thermal, optical, mechanical, etc.—that make them promising candidates for many scientific researches and applications [1]. Conducting polymer composites can be formed by introducing a secondary organic/inorganic component so that enhanced properties—compared to individual components—can be obtained by a combined interaction between the organic/inorganic component and the host polymer matrix [2]. Copolymerization can be used to modify physical properties and overcome drawbacks of individual polymers. Several conducting copolymer composites showed improved properties in many applications. Nano-structured copolymer of aniline (ANI) and pyrrole (Py), (poly(ANI-co-Py)), synthesized at low temperature showed enhanced and selective ammonia sensing behavior as compared with individual polymers PANI and PPy [3]. Zeolite-based nickel-deposited poly(Py-co-fluoro-ANI)/CuS [4] and nano-sulfur/poly(Py-co-ANI) [5] were used as efficient catalysts in sulfur fuel cell applications. Poly(ANI-co-Py)/graphene oxide [6] and transition metal doped poly(ANI-co-Py)/multi-walled carbon nanotube nanocomposite [7] were employed as high performance supercapacitor electrode materials. Zinc-modified poly(ANI-co-Py) coatings showed excellent anti-corrosive performance on low nickel stainless steel [8]. Interconnected poly(ANI-co-Py) nanofibers showed efficient removal of organic pollutants such as Congo red (CR) dye [9], as well as inorganic contaminants such as Cr(IV) ions [10] from aqueous solutions by adsorption.

Congo red, a benzidine-based anionic diazo-dye, constitutes a major common component in colored wastewater resulted from many textile industries [11]. The removal of such pollutants is very important as their presence causes harmful effects to the environment and to humans. Various methods can be employed in the discharging process such as chemical oxidation, electrochemical method, coagulation, ion exchange, and adsorption. Among these methods, adsorption offers the advantages of simplicity, effectiveness, and low cost [12]. Several materials were reported in literature as sorbents for organic dyes [13,14,15,16,17,18], such as conducting polymers [19,20] which are non-toxic, stable under many environmental conditions, can be synthesized in large scale and contain functional groups that promote them as efficient sorbents. Various PANI [21,22,23,24,25,26,27,28,29,30,31], PPy [32,33,34,35], and poly(ANI-co-Py) [9] nano-composites are employed for the removal of organic dyes from aqueous solutions. However, the effect of the starting monomer ratio on the sorption properties has not yet been discussed.

In this work, nano-conducting copolymer composites are prepared by the chemical oxidation of two monomers, ANI and Py, with ferric chloride oxidant in the presence of SiO_2_ nano-paticles and CTAB surfactant. The effect of the starting monomer ratio on structural, surface, and thermal properties is investigated and compared with those properties of individual polymer composites. Prepared nano-conducting polymer composites are applied for the first time for the removal of Congo red from aqueous solutions by adsorption. A proper adsorption isotherm is presented and maximum adsorption capacity values are calculated. These values are discussed and elucidated based on the starting monomer ratio effect on the composite characterizations. In our previous study [36], the effect of the nano-inorganic oxide type in the composite on its sorption capacity for CR has been examined. It is found that nano-silica/PANI composite has a moderate sorption capacity, so it has been chosen in this study to show the enhancement caused by the copolymerization.

## 2. Results and Discussion

### 2.1. Characterizations of Nano-Copolymer Composites

#### 2.1.1. X-ray Diffraction Structural Characterization

**Figure 1:**
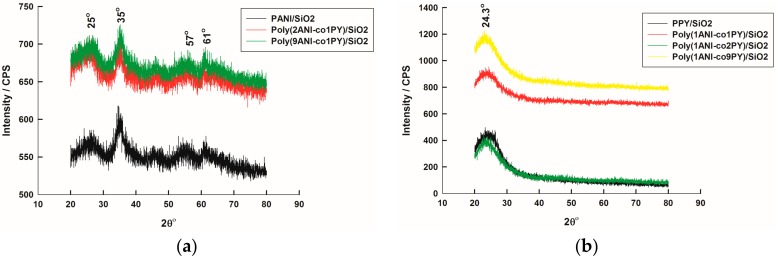
X-ray diffraction (XRD) spectra of homo-polyaniline (PANI), polypyrrole (PPy) and different copolymer of aniline (ANI) and pyrrole (Py) (poly(ANI-co-Py)) composites with SiO_2_; peaks of PANI (**a**) and PPy (**b**) are indexed.

Figure 1 shows X-ray Diffraction (XRD) patterns of homo-PANI and PPy composites with SiO_2_ together with its copolymer composites with SiO_2_. Poly(ANI-co-Py) composites are prepared in different monomer ratios: 1:1, 1:2, 2:1, 1:9, and 9:1.

Table 1 summarizes 2θ values of constituting components of homo- and copolymer composites, SiO_2_, PANI, and PPy. It can be observed that a broad peak appears at around 2θ = 26° [37], for all samples, with an intensity lower than 100 which ascertains the presence of hexagonal amorphous SiO_2_ in all composites as a secondary phase. PANI constitutes the major phase in homopolymer composite with SiO_2_, PANI/SiO_2_, and in poly(2ANI-co-1Py)/SiO_2_ and poly(9ANI-co-1Py)/SiO_2_ samples since its major peak, at 2θ value of 35°, has an intensity value of 100 [38,39], as shown in Figure 1a. On the other hand, PPy is the major phase in homopolymer composite with SiO_2_, PPy/SiO_2_, and in poly(1ANI-co-1Py)/SiO_2_, poly(1ANI-co-2Py)/SiO_2_, and poly(1ANI-co-9Py)/SiO_2_ samples since the major peak (intensity of 100) belongs to PPy at 2θ value of 24.3°, as shown in Figure 1b. It was reported previously that the XRD spectrum of the amorphous PPy has a characteristic single broad peak at around 2θ value of 25° [40]. This slight change in 2θ value may be due to the formation of a composite with SiO_2_ [41].

It can be observed that all copolymer composites have higher peak intensities than those of homopolymer composite which indicates the increase in crystallinity of composites upon copolymerization. In addition, in all copolymer composite samples, two polymers, PANI and PPy, exist. However, in poly(2ANI-co-1Py)/SiO_2_ and poly(1ANI-co-2Py)/SiO_2_, only one polymer phase is present which is PANI or PPy, respectively; this suggests the formation of a copolymer phase in which the absent polymer component cannot be identified (random or branched copolymers).

The Brunauer, Emmett, and Teller (BET) surface area values of all prepared samples, homo- and copolymer composites, are listed in Table 1. It can be seen that the copolymerization decreases the surface area values as compared with the surface area values of individual polymer composites, PANI/SiO_2_ and PPy/SiO_2_. This reduction is more pronounced for comparable starting monomer ratios, i.e., using the starting (1:1) monomer ratio in the synthesis results in the copolymer composite with the lowest surface area, followed by the 1:2 ratio and finally the 1:9 ratio results in the largest surface area; however, in all cases, the surface area is still smaller than that of homopolymer composites. A possible reason for the reduction in surface area values is the core-shell structure, with silica core, that results in a larger particle size and in turn a lower surface area. 

#### 2.1.2. Surface Characterizations by Transmission and Scanning Electron Microscopes

**Figure 2:**
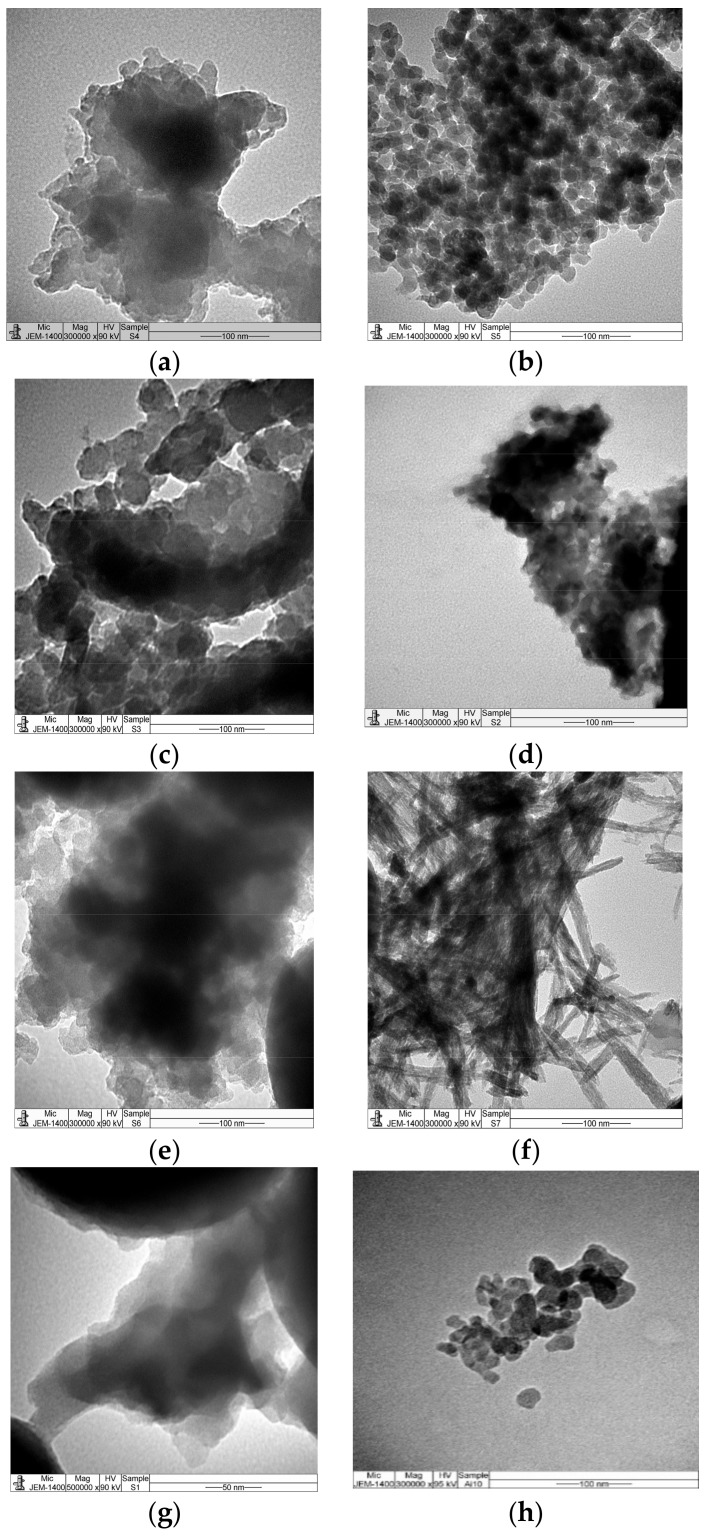
Transmission electron microscopy (TEM) images of PPy/SiO_2_ (**a**); PANI/SiO_2_ (**b**), and different poly(ANI-co-Py) composites in the monomer ratios of 1:9 (**c**); 9:1 (**d**); 1:2 (**e**); 2:1 (**f**); 1:1 (**g**); and silica (**h**).

Transmission electron microscope (TEM) images of PANI, PPy, and different copolymer composites with SiO_2_ are shown in Figure 2a–g. The morphology of PPy/SiO_2_ composite has a characteristic cauliflower shape, Figure 2a. Similar morphology can be shown by poly(1ANI-co-9Py)/SiO_2_, poly(1ANI-co-2Py)/SiO_2_, and poly(1ANI-co-1Py)/SiO_2_ samples, as shown in Figure 2c,e,g, respectively. This agrees with XRD results, where the major constituting phase in these samples is PPy. On the other hand, hexagonal-shaped particles of PANI/SiO_2_ composite can be clearly shown in Figure 2b,d, assigned to the poly(9ANI-co-1Py)/SiO_2_ sample. It can be observed that the poly(2ANI-co-1Py)/SiO_2_ sample, Figure 2f, consists of unique needle-shaped, 1D nano-fiber particles, instead of the expected hexagonal-shaped particles. The preparation of PANI nano-fibers has been successively done by Kaner et al. [42], through the conventional chemical oxidative polymerization of ANI. It was reported that the use of PANI nano-fibers or their composites can significantly enhance the catalytic and sensing properties due to the self-stabilization of nano-fibers by electrostatic repulsions. As a conclusion, it is possible to control the morphology of the copolymer composite by changing the starting monomer ratio.

**Figure 3:**
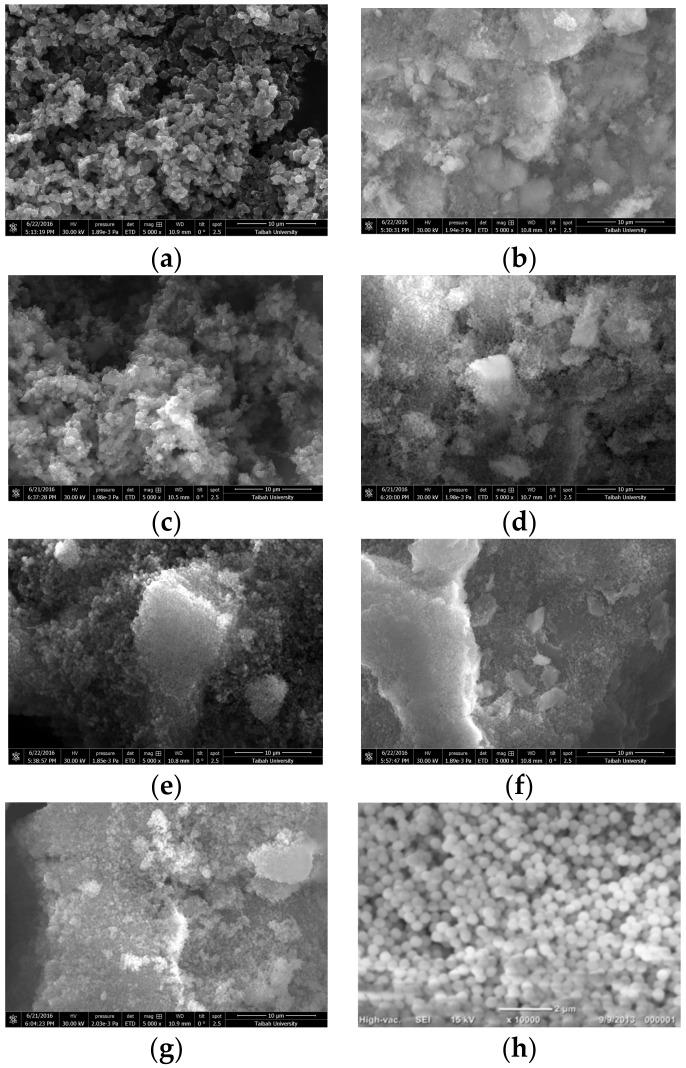
Scanning electron microscope (SEM) images of PPy/SiO_2_ (**a**); PANI/SiO_2_ (**b**), and different poly(ANI-co-Py) composites in the monomer ratios of 1:9 (**c**); 9:1 (**d**); 1:2 (**e**); 2:1 (**f**); 1:1 (**g**); and silica (**h**).

Scanning electron microscope (SEM) photos for PANI, PPy, and different copolymer composites with SiO_2_ are shown in Figure 3. For PPy/SiO_2_ composite, the compact cauliflower morphology can be seen, while in case of silica phase, it cannot be identified from the SEM image, as shown in Figure 3a. The same morphology is assigned to the poly(1ANI-co-9Py)/SiO_2_ sample, as shown in Figure 3c. On the other hand, the morphology can be distinguished between the compact polymeric phase and spherical SiO_2_ grains for PANI/SiO_2_ composite, as shown in Figure 3b, and for poly(9ANI-co-1Py)/SiO_2_, poly(2ANI-co-1Py)/SiO_2_, poly(1ANI-co-2Py)/SiO_2_, and poly(1ANI-co-1Py)/SiO_2_ samples, in which ANI has a major or comparable amount to Py in the starting monomer ratio, as shown in Figure 3d–g, respectively.

#### 2.1.3. Thermal Analysis

**Figure 4:**
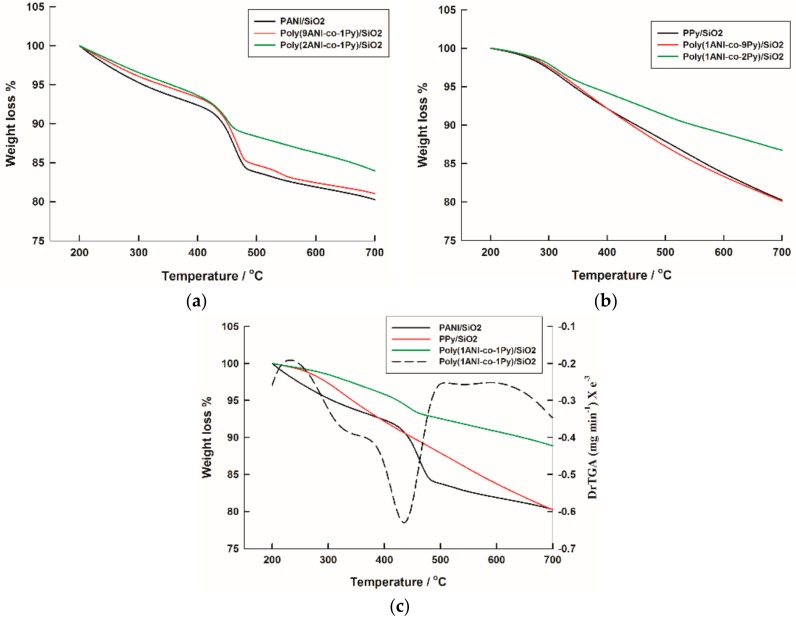
Thermal gravimetric analysis (TGA) curves of (**a**) PANI, poly(9ANI-co-1Py), and poly(2ANI-co-1Py); (**b**) PPy, poly(1ANI-co-9Py), and poly(1ANI-co-2Py); and (**c**) PANI, PPy, and poly(1ANI-co-1Py) composites with SiO_2_.

The thermal behavior of pre-dried homo- and copolymer composites in the temperature range of 200–700 °C, is presented in Figure 4; for PANI/SiO_2_ composite, it is shown in Figure 4a. The first stage of degradation, before 400 °C, corresponds to the degradation of low molecular weight polymeric chains. At temperatures above 400 °C, the polymer completely decomposes with a weight loss of 16% [43]. The residual is assigned to SiO_2_ which is characterized by its high thermal stability [44]. The poly(9ANI-co-1Py)/SiO_2_ and poly(2ANI-co-1Py)/SiO_2_ samples offer exactly the same thermal behavior as homo-PANI composite with relatively higher thermal stabilities as indicated by the reduced weight loss values of 15% and 11%, respectively. For PPy/SiO_2_ composite, as shown in Figure 4b, the degradation of the polymer chain starts at temperature above 250 °C [45] with a 20% weight loss. An identical TGA curve is assigned to the poly(1ANI-co-9Py)/SiO_2_ sample, while very similar behavior with a reduced weight loss of 13% is shown by the poly(1ANI-co-2Py)/SiO_2_ sample. The poly(1ANI-co-1Py)/SiO_2_ sample, as shown in Figure 4c, shows a combined thermal behavior for which two degradation steps can be observed, the first at temperature above 250 °C with a weight loss of 2% and the second stage starts above 400 °C with a weight loss of 7%; that is to say, a total polymer percent of 9%. It can be concluded that the copolymerization enhances the thermal stability and this effect is more pronounced by using comparable starting monomer ratios. In other words, the highest thermal stability is assigned to the poly(1ANI-co-1Py)/SiO_2_ sample, prepared by using an equal starting monomer ratio.

#### 2.1.4. Fourier Transform Infrared Spectroscopy Structural Characterization

**Figure 5:**
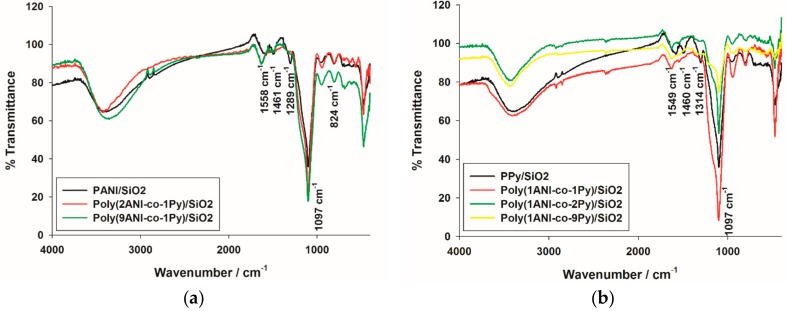
Fourier transform infrared spectroscopy (FTIR) spectra of (**a**) PANI, poly(2ANI-co-1Py), and poly(9ANI-co-1Py); and (**b**) PPy, poly(1ANI-co-1Py), poly(1ANI-co-2Py), and poly(1ANI-co-9Py) composites with SiO_2_.

Fourier transform infrared spectroscopy (FTIR) spectra of PANI and PPy and their different copolymer composites with SiO_2_ are shown in Figure 5. It has been reported that the FTIR spectrum of PANI has peaks at 1558 and 1461 cm^−1^ assigned to the C=N and C=C stretching of quinoid and benzenoid rings, respectively and peaks at 1289 and 824 cm^−1^ for the stretching of C–N and bending of C–H (out of plane) in the benzene ring, respectively [26]. However, the FTIR spectrum of PPy consists of peaks at 1549 and 1460 cm^−1^ assigned to asymmetric and symmetric C–C stretching vibrations of the pyrrole ring, at 1314 cm^−1^ for C–N stretching vibration and at 1050 cm^−1^ for bending vibration of the C–H bond in the pyrrole ring [35]. It can be seen in Figure 5a that PANI/SiO_2_, poly(2ANI-co-1Py)/SiO_2_, and poly(9ANI-co-1Py)/SiO_2_ samples have identical FTIR spectra that contain all the characteristic PANI peaks with a slight shift due to the composite formation with SiO_2_; this agrees with the fact that PANI constitutes the major phase in poly(2ANI-co-1Py)/SiO_2_, and poly(9ANI-co-1Py)/SiO_2_ samples. On the other hand, PPy/SiO_2_, poly(1ANI-co-1Py)/SiO_2,_ poly(1ANI-co-2Py)/SiO_2_, and poly(1ANI-co-9Py)/SiO_2_ samples have similar FTIR spectra that consist of the PPy characteristic peaks. In addition, a peak at 1090–1110 cm^−1^ appears in all homo- and copolymer composites; this peak is assigned to Si–O–Si stretching vibrations which ascertains the presence of SiO_2_ in all samples [46].

### 2.2. Nano-Copolymer Composites as Sorbents for CR Removal

In this section, the prepared nano-poly(ANI-co-Py) composites with SiO_2_ are employed as sorbents for the removal of CR dye from aqueous solutions. The copolymerization effect on the sorption capacity, with different starting monomer ratios is investigated by performing the adsorption test with different initial dye concentrations ranging from 5 to 100 ppm. Then, proper adsorption isotherm models are tested to analyze the adsorption data.

Langmuir and Fruendlich isotherms are applied to the data of CR adsorption onto different nano-composites. The Langmuir isotherm describes the adsorption of adsorbate on homogeneous adsorbent. It explains the monolayer adsorption where there are no interactions between the adsorbate molecules. The linear equation of the Langmuir model can be represented by [47]:

(1) $$\frac{C_{e}}{q_{e}}=\frac{1}{q_{m}K_{L}}+\frac{C_{e}}{q_{m}}$$

where *q_m_* is the maximum amount sorbed (mg g^−1^) when the monolayer is complete. *K_L_* is the Langmuir constant which is related to the energy of the adsorption (L mg^−1^).

For dimensionless constant, *R_L_* can be defined as follows:

(2) $$R_{L}=\frac{1}{1+K_{L}C_{i}}$$

where *K_L_* is the Langmuir constant, *C_i_* is the initial dye concentration (mg L^−1^). *R_L_* value indicates that the Langmuir isotherm is favorable (0 < *R_L_* <1), unfavorable (*R_L_* > 1), linear (*R_L_* = 1) and irreversible (*R_L_* = 0) [48].

The Freundlich isotherm describes the adsorption of adsorbate on a heterogeneous adsorbent. The linear equation of the Freundlich model is given as [48]:

(3) $$\ln q_{e}=\ln K_{f}+\frac{1}{n}\ln C_{e}$$

where *K_f_* and *n* are isotherm constants that indicate the adsorption capacity and intensity of the adsorption, respectively.

**Figure 6:**
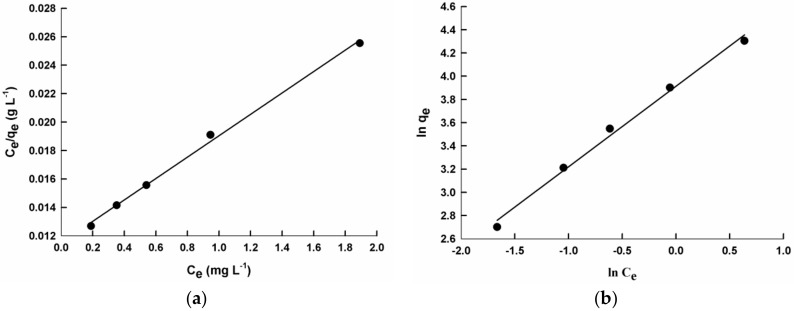
Langmuir (**a**) and Freundlich (**b**) isotherms for Congo red (CR) adsorption onto poly(2ANI-co-1Py)/SiO_2_ composite.

Figure 6 shows the Langmuir and Freundlich adsorption isotherms of poly(2ANI-co-1Py) composite with SiO_2_ for CR, as an example. Langmuir and Freundlich adsorption parameters for PANI/SiO_2_, PPy/SiO_2_ and different copolymer composites are calculated and listed in Table 2.

According to the correlation coefficient values (*r*^2^) of both isotherms, as shown in Table 2, it can be concluded that the Langmuir isotherm fits the adsorption data better than the Freundlich isotherm (*r*^2^ values are closer to unity in the case of the former). Therefore, the adsorption of CR on PANI/SiO_2_, PPy/SiO_2_, and its different copolymer composites occurs at homogeneous adsorption sites to form the adsorbate monolayer. The maximum adsorption capacity values are calculated and listed in Table 2. It can be seen that PPy/SiO_2_ composite has a larger adsorption capacity than that of PANI/SiO_2_ composite; *q_m_* values are 90.9 and 50.0 mg g^−1^ which may be due to its larger surface area of 122.8 and 72.4 m^2^ g^−1^, respectively. The copolymerization decreases the sorption efficiency of PPy/SiO_2_ composite, except for the poly(2ANI-co-1Py)/SiO_2_ sample, the *q_m_* value of which is 142.9 mg g^−1^. However, the copolymerization increases the sorption efficiency of PANI/SiO_2_ composite, except for the poly(1ANI-co-2Py)/SiO_2_ sample, the *q_m_* value of which is 41.7 mg g^−1^. It is worth mentioning that the poly(2AN-co-1Py)/SiO_2_ sample has superior sorption capacity compared to the homopolymer and other copolymer composites, the *q_m_* value of which is 142.9 mg g^−1^. This can be explained by its unique morphology of 1D nano-fibers, which results in enhanced sorption properties [42].

Therefore, the starting monomer ratio, used during the synthesis, affects not only each monomer composition in the copolymer but also the copolymer surface area, and morphology, as well as the polymeric phase amount in the composite, as indicated by the TGA results. All these effects mean that the starting monomer ratio is an important factor in the evaluation of the sorption ability of the copolymer composite.

## 3. Materials and Methods

### 3.1. Chemicals

Tetraethylorthosilicate, ANI (99.5%), Py (98%), ferric chloride, sulfuric acid (95–97%), absolute ethanol (99.8%), ammonium hydroxide (33%), and cetyltrimethylammonium bromide (CTAB) are purchased from Sigma Aldrich. CR (C_32_H_22_N_6_Na_2_O_6_S_2_) was bought from (Brixworth, Northants, UK). All solutions were prepared by double distilled water.

### 3.2. Preparation of Nano-Silica

Silica nano-particles can be prepared by the hydrolysis of tetraethylorthosilicate in ethanol medium in the presence of ammonium hydroxide based on the method reported by Rao et al. [46]. An amount of 10.8 mL of water is first added into 58 mL of ethanol and stirred for 10 min. An amount of 1.6 mL of tetraethylorthosilicate is then added and again stirred for 20 min. An amount of 2.6 mL ammonium hydroxide is added as a catalyst to promote the condensation reaction. The mixture is stirred for 1 h to obtain a white turbid SiO_2_ solution.

### 3.3. Preparation of Nano-Copolymer Composites

Nano-copolymer composites have been chemically prepared by the oxidative copolymerization of ANI and Py in sulfuric acid as a dopant in the presence of nano-silica particles dispersion. Nano-silica is first dispersed in 10^−3^ mol L^−1^ CTAB solution and sonicated for 30 min. Then, FeCl_3_ dissolved in distilled water is added. While stirring, 0.1 mol L^−1^ of ANI and Py solutions dissolved in 0.1 mol L^−1^ H_2_SO_4_, in different mixed volume ratios—(ANI:Py) 1:1, 1:2, 2:1, 9:1, 1:9—are injected into the solution drop by drop. The ratio of nano-silica/monomers/FeCl_3_ is 1:2:2. After 6 h, nano-composites are filtered and washed several times with distilled water, then placed in an 80 °C oven until dry. CTAB enhances the solubility of Py and improves the adsorption capacity of composites [31].

### 3.4. Adsorption Experiment

CR dye solutions used in adsorption experiments are prepared by diluting the stock solution (1000 mg L^−1^) to required concentrations. The removal of CR by different composites is carried out by adding 0.05 g of the composite into 25 mL of CR solution at pH = 6 (pH meter HI 2210, Hanna instrument, Hanoi, Romania). Then, samples are placed into a shaker water bath (DKZ Series shaking water bath, Shanghai, China) at a constant speed of 135 rpm at room temperature for 24 h. Samples are centrifuged (HeraeusLabfuge200centrifuge, Thermo scientific, Darmstadt, Germany) at 3500 rpm for 1 h. Residual CR concentration is analyzed by using a UV-Vis spectrometer (Evolution 300 UV-VIS, Thermo scientific, London, UK) at λ_ma_ = 498 nm. The percentage of the dye removal can be calculated according to:

(4) $$\mathrm{Removal}\;\%=\frac{C_{0}-C_{e}}{C_{0}}\times 100$$

The amount of CR adsorbed at the equilibrium, *q_e_* (mg g^−1^) on synthesized nano-composites, is calculated by:

(5) $$q_{e}=\frac{\left(C_{0}-C_{e}\right)V}{W}$$

where *C*_0_ is the initial dye concentration of (mg L^−1^) and *C_e_* is the equilibrium dye concentration (mg L^−1^); *V* is the volume of the solution (L) and *W* is the mass of the adsorbent (g).

### 3.5. Characterizations of Nano-Composites

The phase identification of nano-composites is carried out by using X-ray diffractograms (XRD, Shimadzu, XRD-7000, Tokyo, Japan) at 40 kV and 30 mA, using a CuK_α_ incident beam (λ = 0.154 nm); the scanning range of 2θ is set between 20 and 80 degrees.

The surface morphology of nano-samples is observed by using scanning electron microscopy (SEM, Superscan SS-550, Shimadzu, Tokyo, Japan) and transmission electron microscopy (TEM, JEM 1400, JEOL, Peabody, MA, USA).

The functional groups of nano-composites are identified by Fourier transform infrared spectroscopy (FTIR, IRAffinity-1S, Shimadzu, Tokyo, Japan). TGA is performed after sample drying at 100 °C by using Q600 T.A. Instruments under N_2_ atmosphere at a heating rate of 10 °C min^−1^ to investigate the thermal stability of samples.

## 4. Conclusions

- Nano-conducting copolymer composites of ANI and Py with SiO_2_ have been successively prepared with different starting monomer ratios by the chemical oxidation method in the presence of CTAB.
- PANI is the major phase in PANI/SiO_2_, and in poly(2ANI-co-1Py)/SiO_2_ and poly(9ANI-co-1Py)/SiO_2_ samples; PPy is the major phase in PPy/SiO_2_, and in poly(1ANI-co-1Py)/SiO_2_, poly(1ANI-co-2Py)/SiO_2_, and poly(1ANI-co-9Py)/SiO_2_ samples.
- Copolymerization decreases surface area values and increases the thermal stability as compared with homopolymer composites, PANI/SiO_2_ and PPy/SiO_2_.
- Cauliflower shaped morphology can be observed for PPy/SiO_2_, poly(1ANI-co-9Py)/SiO_2_, poly(1ANI-co-2Py)/SiO_2_, and poly(1ANI-co-1Py)/SiO_2_ samples. On the other hand, hexagonal-shaped particles are assigned to PANI/SiO_2_ and poly(9ANI-co-1Py)/SiO_2_ samples. The poly(2ANI-co-1Py)/SiO_2_ sample offers unique 1D nano-fibers.
- Copolymerization decreases the sorption efficiency of PPy/SiO_2_ composite, except for the poly(2ANI-co-1Py)/SiO_2_ sample; it increases the sorption efficiency of PANI/SiO_2_ composite, except for the poly(1ANI-co-2Py)/SiO_2_ sample. The poly(2AN-co-1Py)/SiO_2_ sample has superior sorption capacity compared to homopolymer and other copolymer composites; the *q_m_* value is 142.9 mg g^−1^ due to its unique morphology which results in enhanced sorption properties.

## Figures and Tables

**Table 1:** 2θ, peak intensity values and Brunauer, Emmett, and Teller (BET) surface areas of constituting homo-polyaniline (PANI), polypyrrole (PPy) and copolymers, (poly(ANI-co-Py)) with different starting monomers ratios, composites with SiO_2_ [36].

		PANI/SiO_2_ Sample		PPy/SiO_2_ Sample		Poly(1ANIco1Py)/SiO_2_ Sample		Poly(1ANIco2Py)/SiO_2_ Sample		Poly(2ANIco1Py)/SiO_2_ Sample		Poly(1ANIco9Py)/SiO_2_ Sample		Poly(9ANIco1Py)/SiO_2_ Sample	
**Standard SiO_2_**															
2θ	I	2θ	I	2θ	I	2θ	I	2θ	I	2θ	I	2θ	I	2θ	I
20	20	20.4	24	-	-	-	-	-	-	-	-	-	-	-	-
26	100	26.0	24	26.0	95	26.1	76	26.1	65	26.1	79	26.3	63	26.0	45
**Standard PANI**															
25	45	25.1	28	-	-	-	-	-	-			-	-	-	-
35	100	35.0	100	-	-	35.2	18	No Peak		34.9	100	34.8	7	35.1	100
57	21	57.0	16	-	-	-	-	-	-			-	-	57.1	14
61	24	60.7	36	-	-	-	-	-	-	60.9	53	-	-	61.0	43
**Standard PPy**															
24.3	100	-	-	24.3	100	24.1	100	24.1	100	No peak		24.3	100	24.5	35
BET surface area/m^2^ g^−1^		72.40		122.81		24.23		47.99		53.69		55.01		68.43	

**Table 2:** Adsorption isotherm constants, maximum adsorption capacity (*q_m_*), Langmuir constant (*K_L_*), and Freundlich constants (*K_f_* and *n*), with correlation coefficient values, *r*^2^, for Congo red (CR) adsorption onto the PANI/SiO_2_, PPy/SiO_2_ and different copolymer composites.

Langmuir Constants		*q_m_* (mg g^−1^)	*K_L_* (L mg^−1^)	*R_L_*	*r*^2^
**PANI/SiO_2_**		50.0	0.190	0.05–0.51	0.981
**PPy/SiO_2_**		90.9	0.096	0.09–0.68	0.998
**Copolymers/SiO_2_ composites**					
ANI	Py	***q_m_* (mg g^−1^)**	***K_L_* (L/mg)**	***R_L_***	***r*^2^**
1	1	83.3	0.285	0.03–0.26	0.986
9	1	62.5	0.592	0.01–0.05	0.999
1	9	83.3	0.260	0.03–0.11	0.996
2	1	142.9	0.636	0.01–0.05	0.997
1	2	41.7	0.235	0.03–0.12	0.998
**Freundlich Constants**		***K_f_* (mg^1−(1/*n*)^L^1/*n*^/g)**	***n***	***r*^2^**	
**PANI/SiO_2_**		7.2	1.58	0.978	
**PPy/SiO_2_**		7.4	1.21	0.993	
**Copolymers/SiO_2_ composites**					
ANI	Py	***K_f_* (mg^1−(1/*n*)^L^1/*n*^/g)**	***n***	***r*^2^**	
1	1	16.2	1.34	0.970	
9	1	21.8	2.01	0.960	
1	9	18.4	1.98	0.920	
2	1	50.1	1.44	0.992	
1	2	13.4	3.70	0.949	
